# Strengthening causal inference from randomised controlled trials of complex interventions

**DOI:** 10.1136/bmjgh-2022-008597

**Published:** 2022-06-09

**Authors:** Jef L Leroy, Edward A Frongillo, Bezawit E Kase, Silvia Alonso, Mario Chen, Ian Dohoo, Lieven Huybregts, Suneetha Kadiyala, Naomi M Saville

**Affiliations:** 1 Poverty, Health, and Nutrition Division, International Food Policy Research Institute, Washington, District of Columbia, USA; 2 Department of Health Promotion, Education, and Behavior, University of South Carolina, Columbia, South Carolina, USA; 3 Animal and Human Health Porgram, International Livestock Research Institute, Nairobi, Kenya; 4 Biostatistics and Data Sciences, FHI 360, Durham, North Carolina, USA; 5 Health Management, University of Prince Edward Island, Charlottetown, Prince Edward Island, Canada; 6 The London School of Hygiene & Tropical Medicine, London, UK; 7 Institute for Global Health, University College London, London, UK

**Keywords:** randomised control trial, intervention study

## Abstract

Researchers conducting randomised controlled trials (RCTs) of complex interventions face design and analytical challenges that are not fully addressed in existing guidelines. Further guidance is needed to help ensure that these trials of complex interventions are conducted to the highest scientific standards while maximising the evidence that can be extracted from each trial. The key challenge is how to manage the multiplicity of outcomes required for the trial while minimising false positive and false negative findings. To address this challenge, we formulate three principles to conduct RCTs: (1) outcomes chosen should be driven by the intent and programme theory of the intervention and should thus be linked to testable hypotheses; (2) outcomes should be adequately powered and (3) researchers must be explicit and fully transparent about all outcomes and hypotheses before the trial is started and when the results are reported. Multiplicity in trials of complex interventions should be managed through careful planning and interpretation rather than through post hoc analytical adjustment. For trials of complex interventions, the distinction between primary and secondary outcomes as defined in current guidelines does not adequately protect against false positive and negative findings. Primary outcomes should be defined as outcomes that are relevant based on the intervention intent and programme theory, declared (ie, registered), and adequately powered. The possibility of confirmatory causal inference is limited to these outcomes. All other outcomes (either undeclared and/or inadequately powered) are secondary and inference relative to these outcomes will be exploratory.

**Summary box**

- Evaluating the impact of complex interventions requires assessing the effectiveness of the intervention on multiple outcomes.
- Researchers conducting randomised controlled trials of these interventions are faced with design and analytic challenges that are not fully addressed in existing guidelines and that are exacerbated by the belief that guidance for trials of non-complex interventions should be strictly applied to trials of complex interventions
- For trials of complex interventions, limiting causal inference to a few primary outcomes is inappropriate, but each primary outcome should be linked to a stated hypothesis, registered before trial start, adequately powered, and fully and transparently reported.
- Inference on undeclared or underpowered outcomes should be considered exploratory.
- Our guidance ensures that trials of complex interventions are conducted to the highest scientific standards while maximising the evidence that can be extracted from each trial.

## Introduction

Effectively addressing global development challenges such as poverty, undernutrition, poor child development and high burdens of infectious and non-communicable diseases requires coordinated multisectoral interventions implemented at different levels. These interventions are typically complex, with multiple intervention components intended to address several underlying and immediate determinants of the problem through multiple paths.

Rigorous evaluations are needed to help build the evidence base on the effectiveness of complex interventions. Among possible evaluation study designs, randomised controlled trials (RCTs) are often preferred because they can mitigate problems of confounding and selection bias.^1^ RCTs are not always appropriate or achievable to evaluate the impact of complex interventions, and evidence from other study designs may be needed to evaluate these interventions.^2–6^ Our study, however, focuses on the use of RCTs to evaluate complex interventions.

The key feature of RCTs is that the intervention is randomly assigned to study participants. When properly implemented, the randomisation is expected, on average, to balance both observed and unobserved participant characteristics across trial arms, thus enabling the attribution of any difference in outcomes across arms to the intervention.^7^ The last decades have seen a rapid rise in the use of the RCT method in fields such as agriculture, nutrition, public health and development economics.^8 9^ With the growing complexity of the interventions that were being evaluated came the need to assess effects across a wide range of impact paths and outcomes. We refer to these trials as RCTs of complex interventions (Box 1).

Box 1 **Efficacy and effectiveness, trials of non-complex and complex interventions, and outcomes: definitions**

An **intervention** is a manipulation of the environment for the purpose of modifying one or more biological, behavioural or social processes and/or endpoints. **Efficacy** is the extent to which an intervention works under ideal and controlled circumstances. The **effectiveness** of an intervention is the degree to which the intervention performs in the real world, that is, under usual circumstances.^64^

**Trials of non-complex interventions** seek to assess the effect of a well-defined single intervention (eg, antibiotic treatment, micronutrient supplement), often on a narrowly defined outcome (eg, number of days ill, micronutrient status). These trials are often based on a solid understanding of the underlying biological mechanisms. Trials of non-complex interventions can be either **efficacy** or **effectiveness** trials. Randomised controlled trial guidance has been developed to avoid the situation in which economic and other interests in showing that an intervention (such as a new drug) works conflict with the integrity of the design, conduct and interpretation of the findings.

**Trials of complex interventions** evaluate the impact of interventions with several interconnected components designed to affect multiple outcomes through one or several mechanisms.^14^ These components can range from behaviour change communication and facilitating women’s empowerment to micronutrient supplements, cash transfers and agricultural extension. Complex interventions intervene in and disrupt the functioning of complex systems by changing relationships, modifying deeply rooted practices, and transforming and redistributing resources.^5^ Trials of complex interventions are typically designed to evaluate **effectiveness**, that is, whether interventions work when used in usual community and facility conditions. The effect of complex interventions often depends on both biological and behavioural mechanisms, but the relative importance of several potential paths of impact is often unknown. Researchers are thus interested in estimating effects on a range of outcomes directly linked to the objectives of the intervention (eg, agricultural productivity, nutritional status, women’s empowerment) and in understanding the underlying mechanisms by documenting effects on intermediate outcomes along the theorised impact paths.

An **outcome** is a phenomenon of theoretical interest that is real, may be observable or latent, and represents the result or consequence of an intervention. A **measure** of an outcome reflects or manifests the outcome.^65^ For example, in an intervention in Costa Rica intended to reduce both food insecurity and excessive body weight,^66^ the main trial outcomes were changes in food insecurity measured by a food-insecurity scale, changes in whole body fatness measured by body mass index, and changes in abdominal fatness measured by waist circumference. Programme impact paths were assessed by assessing impact on several other (intermediate) outcomes.^66^

Important problems with the design, analysis and reporting quality of RCTs have been identified.^10–12^ Although excellent guidance about how to conduct RCTs is available,^7 13 14^ researchers conducting RCTs of complex interventions face challenges in designing and analysing trials that are not fully addressed in existing guidelines and are exacerbated by the belief common among scientists that guidance for trials of non-complex interventions should be strictly applied to trials of complex interventions. Challenges often relate to the number of intermediate and final outcomes that the trialist can assess and how that affects causal inference. These challenges lead to questions and confusion about how to conduct trials of complex interventions and interpret their findings. Our objective is not to review current practice, but to provide guidance that ensures that trials of complex interventions are conducted to the highest scientific standards while maximising the evidence that can be extracted from each trial. We start from basic principles to formulate recommendations.

We discuss how to reduce the probability of false positive findings (reporting an intervention effect when it does not truly exist) and false negative findings (not finding an effect when it does exist in reality). We explain why the selection of outcomes should be based on the programme theory and demonstrate that the recommendation to limit the number of primary outcomes to one^7^ is unnecessary. We clarify why multiplicity in trials of complex interventions is best managed through careful planning and interpretation rather than through post hoc analytical adjustment. Finally, we address other features of importance for these trials and provide recommendations on how to conduct trials of complex interventions. Even though our work was motivated by the need for better guidance to conduct such trials, many of our recommendations are applicable to all RCTs.

## Purpose and use of RCTs of complex interventions

To determine the effectiveness of an intervention, a counterfactual for the intervention arm (ie, an estimate of what the outcome(s) would have been in the absence of the intervention) is needed. Because researchers can only observe outcomes for each study unit—units could be, eg, individuals, households, villages, clinics, of some combination of these—under at most one intervention alternative (eg, each unit can only be assigned to one of the study arms), they must rely on other units to construct the counterfactual. In an RCT, the counterfactual for intervention units is built using the outcomes for comparison units, with random assignment determining whether units receive an intervention or not.^1^

RCTs support inference about causal relationships for two reasons. First, the assignment of the participants to study arms is done by the investigators (rather than being selected by the participants) and outcomes can be tracked to have happened post-intervention, making the direction of causality clear. This benefit of establishing the temporal relationship between exposure to the intervention and the change in outcomes is obtained in any design in which investigators assign the condition, not just randomised designs. Second, properly implemented randomization makes it likely that the comparison arm provides a valid counterfactual and that the arms are equivalent, on average, for all participant characteristics at the beginning of the trial, including characteristics not measured or observable. Randomisation mitigates the potential for confounding and reduces the possibility of selection bias. RCTs prioritise ensuring internal validity and are seen in medicine as the standard for establishing quality clinical evidence.^2^ To be useful as a source of evidence for public policy, RCTs of complex multicomponent interventions must fully embrace this complexity by assessing the impact on all relevant outcomes.^2–6^

Many RCT designs are used to evaluate the effectiveness of complex interventions. The study might assess each individual before and after implementation of the intervention and be longitudinal at the individual level. This design strengthens inference and increases statistical power but is subject to lost to follow-up and potential attrition bias.^15^ Alternatively, the study might assess a new sample of individuals after implementation of the intervention. This repeated cross-sectional design is useful when, for example, children of a specified age range are of interest and the period between baseline and end-line is long. Many trials of complex interventions randomly assign clusters of individuals (or households) rather than individuals to the study arms.^16^ This design is used when the intervention includes components that cannot or should not be directed to individuals or households, such as community-led total sanitation and group-based behaviour change communication. The clustering of individuals or households has implications for the estimation of effects and uncertainty, thus affecting the calculation of the sample size needed. Since observations within study clusters are typically more like one another than they are to observations in other study clusters, cluster sampling usually results in increased variability of estimates compared with simple (ie, individual) sampling of the same size.

Investigators doing complex intervention trials typically assess the effectiveness of the intervention on multiple outcomes because the intervention (1) is complex, with many different components and activities (box 1); (2) is designed and expected to have effects on multiple outcomes, for example, on agriculture productivity, women’s empowerment, mental or physical health, child development and nutrition; and (3) exerts its effects through theorised complex programme impact paths, each of which has intermediate outcomes for which assessing impact is useful. Therefore, trials of complex interventions are conducted often not to simply determine whether the intervention was effective. Instead, the conclusion is typically nuanced, detailing that the intervention improved some outcomes but not others. That is, the effectiveness of complex interventions is generally not simply judged based on either having at least one positive effect (among a set of outcomes specified before the start of the trial) or having a simultaneous positive effect on all important outcomes. Furthermore, recent guidance emphasises that scientists should stop using the concept and term ‘statistically significant’ and declaring a p value of less than 0.05 as strong evidence against a null hypothesis and instead report exact p values or CIs.^17–20^ In trials of complex interventions, the focus is on estimates of effects and estimates of uncertainty about the estimates of effects (eg, exact p values or confidence intervals) rather than on simple binary inferences implied by statistical significance. In sum, judgement about effectiveness in trials of complex interventions requires integration of evidence regarding several outcomes and the confidence in that evidence.

## Accuracy of inference and inadequacy of existing recommendations for trials of complex interventions

Accuracy of inference from RCTs is undermined by false positive and false negative results. A false positive result occurs when we conclude, based on the statistical tests, that the intervention had an effect when it truly did not, a type I error. A false negative result occurs when we fail to identify an intervention effect that truly exists, a type II error. We discuss both types of errors from the perspectives of both any particular outcome and the larger evidence base.

### False positive results

The probability of a false positive result when testing the effect on one specific outcome is typically measured using the p-value from the statistical test. A p value of 0.1 implies that there is 1/10 chance that we identify an intervention effect when there was no true effect. If all trials fully reported all outcomes tested, then the overall probability of false positive results among all estimated effects would be accurately measured by the aggregate p value, but frequently trial reporting is done selectively. Selective reporting of outcomes on which the intervention had a positive effect (and under-reporting of null findings) is well documented and contributes to publication bias.^1 7 21 22^ Null results (ie, the absence of an observed effect), or any observed effects opposite to the desirable one, are much less likely to be written up and published. Not reporting of unfavourable results might be due to possible economic gains (eg, selling a solution to a problem) and academic incentives (eg, it is easier to get a paper published when positive large effects are reported).^23^ When trial reporting is done selectively, based on the study findings and not simply on the evaluation questions the trial sought to answer, our collective understanding of what works is biased. To illustrate the problem, assume that the same intervention is tested in 40 different well conducted and adequately powered trials and that 1 of these trials documents an effect on the outcome of interest. If all trials are fully reported, then the evidence base will show that the effect was observed in only 2.5% of the trials, likely reflecting a false positive result. If only the trial that found a positive effect is reported, however, readers will wrongly conclude from the evidence that the intervention is effective for that outcome.

### False negative results

The ability to find a positive effect on an outcome when it truly occurred depends on the power of the study. Statistical power is one minus the probability of a type II error. A statistical power of 80%, which is frequently used in efficacy and effectiveness trials, leaves a 1/5 chance that an actual effect is not detected. Increasing power to 90% reduces that probability to 1/10. Underpowering a study leaves ambiguous the interpretation of a null result (ie, not finding an effect) because true absence of an effect cannot be separated from the effect being too small to be detected given the limited power.

Underpowering studies also negatively influences how studies contribute to the evidence base. Since underpowered studies can only detect large effects, small effects often remain unreported. Selective reporting of the large effects will bias the average effect in the published literature upward, that is, the average magnitude of the impact documented in papers will be much larger than the true expected effect of the intervention.

### Inadequacy of typical recommendations for trials of complex interventions

To safeguard the accuracy of inference from RCTs, regulatory agencies (eg, US Food and Drug Administration, the European Medicines Agency), research funders (eg, UK Medical Research Council) and groups of trial experts (eg, Consolidated Standards of Reporting Trials or CONSORT) have devised guidelines on how to design, implement and report on RCTs.^7 13 14 24–30^ A key feature of these guidelines, which target trialists in health-related fields, is to declare primary and secondary outcomes (table 1). The distinction between primary and secondary outcomes is uncommon in RCTs conducted in other fields such as economics.^1 31 32^ Another common feature across these guidelines targeted to health researchers is to limit the number of primary outcomes to a few; some guidance states that the number should be one. Guidelines generally agree that primary outcomes relate to the primary study objective, that they need to be prespecified, and that they drive sample size requirements to ensure adequate statistical power. Secondary outcomes are inconsistently defined.

**Table 1:** Definition of primary and secondary outcomes and implications for causal inference in guidance documents

Source	Relevant text from guidance (verbatim citation)	Items included in the definition of primary outcomes	Items included in the definition of secondary outcomes	Statements about causal inference relative to primary and secondary outcomes
FDA Statistical Principles for Clinical Trials^13^	The primary variable (target variable, primary endpoint) should be the variable capable of providing the most clinically relevant and convincing evidence directly related to the primary objective of the trial. There should generally be only one primary variable. The primary variable should generally be the one used when estimating the sample size. The primary variable should be specified in the protocol, along with the rationale for its selection. Redefinition of the primary variable after unblinding will almost always be unacceptable, since the biases this introduces are difficult to assess. Secondary variables are either supportive measurements related to the primary objective or measurements of effects related to the secondary objectives. Their predefinition in the protocol is also important, as well as an explanation of their relative importance and roles in interpretation of trial results. The number of secondary variables should be limited and should be related to the limited no of questions to be answered in the trial	**Included in definition** Most clinically relevant/related to primary objective Prespecified Determines sample size Number of primary outcomes: 1	**Included in definition** Supportive of primary objective, related to secondary objective Prespecified Number of secondary outcomes: Limited **Not included in definition** Determines sample size	Language does not suggest that causal inference depends on primary or secondary designation: For example, ‘Secondary variables are either supportive measurements related to the primary objective or measurements of **effects** related to the secondary objectives.’
FDA Multiple Endpoints in Clinical Trials^24^	Positive results on the secondary endpoints can be interpreted only if there is first a demonstration of a treatment effect on the primary endpoint family	No definition provided	No definition provided	Causal inference with respect to secondary outcomes depends on finding a positive effect on primary outcomes: ‘econdary endpoints maybe selected to demonstrate additional **effects** after success on the primary endpoint.’
EU Guideline for good clinical practice^25^	A primary endpoint(s) should reflect clinically relevant effects and is typically selected based on the principal objective of the study. Secondary endpoints assess other drug effects that may or may not be related to the primary endpoint. Endpoints and the plan for their analysis should be prospectively specified in the protocol. A surrogate endpoint is an endpoint that is intended to relate to a clinically important outcome but does not in itself measure a clinical benefit. Surrogate endpoints may be used as primary endpoints when appropriate (when the surrogate is reasonably likely or well known to predict clinical outcome). The methods used to make the measurements of the endpoints, both subjective and objective, should be validated and meet appropriate standards for accuracy, precision, reproducibility, reliability, and responsiveness (sensitivity to change over time). Statistical assessments of sample size should be based on the expected magnitude of the treatment effect, the variability of the data, the specified (small) probability of error (see ICH E9) and the desire for information on subsets of the population or secondary endpoints.	**Included in definition** Most clinically relevant/related to primary objective Prespecified Determines sample size **Not included in definition** Number of primary outcomes	**Included in definition** Supportive of primary objective, related to secondary objective Prespecified **Not included in definition** Determines sample size Number of secondary outcomes	Language does not suggest that causal inference depends on primary or secondary designation: For example, ‘Secondary endpoints assess other drug **effects** that may or may not be related to the primary endpoint.’
Explanation and elaboration: updated guidelines for reporting parallel group randomised trials^7^	The primary outcome measure is the prespecified outcome considered to be of greatest importance to relevant stakeholders (such a patients, policy-makers, clinicians, funders) and is usually the one used in the sample size calculation. Some trials may have more than one primary outcome. Having several primary outcomes, however, incurs the problems of interpretation associated with multiplicity of analyses and is not recommended. Primary outcomes should be explicitly indicated as such in the report of an RCT. Other outcomes of interest are secondary outcomes (additional outcomes). There may be several secondary outcomes, which often include unanticipated or unintended effects of the intervention, although harms should always be viewed as important whether they are labelled primary or secondary. All outcome measures, whether primary or secondary, should be identified and completely defined.	**Included in definition** Most clinically relevant/related to primary objective Prespecified Determines sample size Number of primary outcomes: 1 recommended	**Included in definition** Supportive of primary objective, related to secondary objective (unintended effects) Prespecified Number of secondary outcomes: several **Not included in definition** Determines sample size	Language does not suggest that causal inference depends on primary or secondary designation: ‘There may be several secondary outcomes, which often include unanticipated or unintended **effects** of the intervention…’
Developing and evaluating complex interventions: the new Medical Research Council guidance^14 26^	A single primary outcome and a small number of secondary outcomes are the most straight forward for statistical analysis but may not represent the best use of the data or provide an adequate assessment of the success or otherwise of an intervention that has effects across a range of domains. A good theoretical understanding of the intervention, derived from careful development work, is key to choosing suitable outcome measures. The primary outcome should be clearly identified. Where interventions have a diverse range of outcomes, it is preferable to combine them into a single generic measure such as the quality-adjusted life-year. Such measures also enable comparisons across cost-effectiveness studies.	**Included in definition** Number of primary outcomes: several possible, but preferably combined into one measure **Not included in definition** Most clinically relevant/related to primary objective Prespecified Determines sample size	**Included in definition** Number of secondary outcomes: small no **Not included in definition** Supportive of primary objective, related to secondary objective Prespecified Determines sample size	Nothing on causal inference
The REFLECT Statement: Reporting Guidelines for Randomised Controlled Trials in Livestock and Food Safety: Explanation and Elaboration^27^	Primary outcome The primary outcome refers to an outcome variable of interest, the expected value of which is used to determine the study sample size. If researchers have more than one outcome of interest, the sample size will be determined by the outcome that needs the highest sample size, and this will be the primary outcome. Secondary outcome(s) This refers to another outcome measure that is potentially equally important but not used to determine the sample size. There may be more than one secondary outcome.	**Included in definition** Most clinically relevant/related to primary objective Determines sample size Number of primary outcomes: 1 **Not included in definition** Prespecified	**Included in definition** Supportive of primary objective, related to secondary objective Number of secondary outcomes: several Determines sample size: No **Not included in definition** Prespecified	Language does not suggest that causal inference depends on primary or secondary designation.
Best Practices for Food-Based Clinical Trials - Guidance for Planning, Conducting and Reporting on Human Studies to Support Health Claims^28^	The primary outcome is used to answer the principal research question and to calculate sample size. The primary outcome should be well defined and reliable for assessing important aspects of health, sensitive to the effect of the intervention, and measurable and interpretable. Trials may have additional outcomes to measure different aspects of the intervention effect, known as secondary or tertiary outcomes. However, a prior in sample size is generally calculated to determine power for primary outcomes; therefore, if the study includes secondary and tertiary outcomes, it is important to ensure the sample size can adequately investigate the impact of additional outcomes.	**Included in definition** Most clinically relevant/related to primary objective Determines sample size Prespecified Number of primary outcomes: 1 (implied)	**Included in definition** Determines sample size **Not included in definition** Supportive of primary objective, related to secondary objective Prespecified Number of secondary outcomes	Language does not suggest that causal inference depends on primary or secondary designation: For example, ‘if the study includes secondary and tertiary outcomes, it is important to ensure the sample size can adequately investigate the **impact** of additional outcomes’
Clinical Trials Registration and Results Information Submission; Final Rule^29^	**Primary outcome measure information** ‘Primary outcome measure’ means the outcome measure(s) of greatest importance specified in the protocol, usually the one(s) used in the power calculation. Most clinical studies have one primary outcome measure, but a clinical study may have more than one. **Secondary outcome measure information** ‘Secondary outcome measure’ means an outcome measure that is of lesser importance than a primary outcome measure, but is part of a prespecified analysis plan for evaluating the effects of the intervention or interventions under investigation in a clinical study and is not specified as an exploratory or other measure. A clinical study may have more than one secondary outcome measure. **Other prespecified outcome measures** Definition: Any other measurements, excluding post hoc measures, that will be used to evaluate the intervention(s) or, for observational studies, that are a focus of the study.	**Included in definition** Most clinically relevant/related to primary objective Prespecified Determines sample size Number of primary outcomes: 1 (mostly)	**Included in definition** Supportive of primary objective, related to secondary objective Prespecified Number of secondary outcomes: several **Not included in definition** Determines sample size	Nothing on causal inference

EU, European Union; FDA, Food and Drug Administration; ICH, International Council for Harmonisation of Technical Requirements for Registration of Pharmaceuticals for Human Use; RCT, randomised controlled trial.

The recommendation to limit the number of outcomes, especially primary outcomes, stems from concern about a higher probability of false positive findings with a larger number of outcomes, which is transformed into concern about multiplicity. We discuss below that multiplicity is typically not a threat to the accuracy of inference from RCTs of complex interventions. The appropriate strategy to help minimise the problem of false positive findings is to declare and register outcomes in advance, use one measure per outcome and report on all outcomes irrespective of whether an intervention effect was found. Excessive limiting of the number of outcomes comes at an unacceptable cost as it restricts what can be learnt from the evaluation of a complex intervention by unnecessarily forcing researchers to not assess the intervention’s effect on outcomes important to decision makers and on outcomes along the impact paths.

The guidance to prespecify outcomes implies that outcomes are declared and registered in a public trial registry before the trial is started. Registration ensures transparency about the preplanned outcome(s) purported to represent whether the intervention worked as intended and helps to address the problems of false positive findings and selective reporting. The recommendation to adequately power all primary outcomes reduces the probability of false negative findings.

The formal guidelines have been supplemented with publications in the scientific literature that describe how the type of outcome determines the type of inference.^33 34^ Based on typical recommendations, common thinking among many trialists, including many complex intervention trialists, can be summarised as (1) only primary outcomes allow the researcher to draw confirmatory (ie, causal) conclusions and (2) conclusions based on the secondary outcomes are exploratory and not confirmatory. This belief, however, is not supported by the existing guidelines and does not logically follow from the attributes of primary and secondary outcomes as described in these guidelines. The strength of the causal inference for any specific outcome depends on the study design and implementation and not on the label given to the outcome. The strength of a well-designed and properly implemented RCT lies in the ability to conclude that the observed effect on an outcome is due to the intervention and not to something else. The ability to reach this causal conclusion is the same for each trial outcome, irrespective of the number of outcomes, the label the researcher assigned to the outcome (ie, primary or secondary), and whether the outcome was declared before the trial or after the trial was concluded. What matters is the accuracy of the inference that can be made from the perspective of the larger evidence base. That is, what is the probability that some of the observed effects are false positive findings, are the effects estimated with confidence only the ones that are exceptionally large due to limited statistical power, and what is the probability that not finding an effect reflects the true absence of an effect? Whether causal inference is confirmatory or exploratory can only be determined relative to the larger evidence base and not with respect to any individual outcome. That is, confirmatory and exploratory are properties of the inference and not of specific outcomes.

In summary, current guidance and recommendations address many but not all the concerns related to false positive and false negative findings. Simply classifying outcomes as primary and secondary does not sufficiently protect against these two types of false findings. Limiting the number of outcomes to just a few unnecessarily restricts what can be learnt from RCTs of complex interventions. The number of outcomes is discussed later in the manuscript.

## Typology of outcomes and causal inference

For trials of complex interventions, the distinction between primary and secondary outcomes as defined in current guidelines is not useful. In these trials, primary outcomes should be defined as those that are relevant based on the intervention intent and programme theory, declared (ie, registered), and adequately powered. Primary outcomes can include both intervention endpoints and intermediary outcomes. Confirmatory causal inference is limited to primary outcomes (table 2). Secondary outcomes are all other outcomes, that is, those that are either undeclared and/or inadequately powered. Inference on these outcomes will be exploratory.^35^ The distinction between primary and secondary outcomes is based on three basic principles presented in the next section.

**Table 2:** Outcomes, effects and inference.

Type of outcome		Primary or secondary	Inference	
			Was an effect found?	
			Yes	No
Declared, registered	Adequately powered	Primary*	Confirmatory *‘Intervention X has an effect on outcome Y’*	Confirmatory *‘Intervention X does not have an effect of the magnitude for which the trial was powered on outcome Y’*
	Inadequately powered	Secondary	Exploratory *‘Intervention X has* an effe*ct on outcome Y, but the magnitude of the estimated effect is likely to be larger than the average population effect’*	No conclusion possible
Undeclared, not registered	Adequately powered	Secondary	Exploratory *‘Intervention X may have an effect on outcome Y’*	Exploratory *‘Intervention X may not have an effect of the magnitude for which the trial was powered on outcome Y’*
	Inadequately powered	Secondary	Exploratory *‘Intervention X may have an effect on outcome Y, but the magnitude of the estimated effect is likely to be larger than the average population effect’*	No conclusion possible

*Primary outcomes are those that are relevant based on the intervention intent and programme theory and therefore declared and adequately powered. Secondary outcomes are all other outcomes, including those either undeclared and/or inadequately powered.

## Basic principles for trials of complex interventions

Given that some typical recommendations are not appropriate for trials of complex interventions, building appropriate recommendations from basic principles is needed. To mitigate threats to the accuracy of inference from complex intervention trials, researchers should adhere to three principles.

First, the **selection of outcomes should be driven by the intent of the intervention and its programme theory**. The programme theory (also referred to as theoretical model or theory of change) explains how the intervention is understood to result in or contribute to a chain of actions and their consequences that produce the intended or actual impacts.^36^ Articulating the programme theory is important to generate testable hypotheses for the empirical analyses and enable interpretation and application of empirical results to programming and policy in other contexts. A programme theory framework (also known as diagram of programme impact paths or causal diagram) identifies the key programme components included in a programme, what may affect optimal delivery or utilisation of each component, the assumptions associated with each of the components, and how the components are expected to be linked to achieve impact. The paths to impact (also referred to as causal mechanisms) show explicitly how intervention components are hypothesised to affect proximal outcomes and how these outcomes may subsequently change more distal outcomes.^14 31 37 38^ Using the programme impact paths, all outcomes the programme is designed or expected to affect are identified. The list of outcomes should not be limited to the endpoints. Imagine an agricultural livelihood intervention combined with behaviour change communication targeted to women that seeks to improve diet during pregnancy. The list of identified outcomes should not only include pregnant women’s dietary adequacy but should also consider intermediate outcomes such as agriculture production, household food expenditure and consumption, household food security and women’s knowledge about dietary requirements during pregnancy. If no effects are observed in these intermediary outcomes, it is unlikely that improvements will be found in women’s diets. Conversely if positive effects, attributable to the programme, are seen in all outcomes along the impact path, plausibility that the impacts are due to the programme is strengthened. The list should not be limited to positive or desirable outcomes. The intervention may, for instance, benefit others in the household, could negatively affect women’s status, increase conflict, or lead to excessive weight gain. Researchers should attempt to also include these potentially negative outcomes in their study. Finally, evaluating the impact on all relevant outcomes is necessary to accurately quantify the full return on programme investment.^39^

Only outcomes that are relevant and are intended to be analysed and published should be included. Relevant means that the intervention can be expected to affect the outcome (either positively or negatively) and that the findings are expected to meaningfully contribute to our understanding of whether and how interventions work. Additional, irrelevant outcomes increase participant burden and require more funding, hence the moral obligation to only include relevant outcomes and commit to reporting all outcomes included in the study. Finally, only one outcome measure per outcome should be included to avoid selective reporting of the measure for which an impact is found.

Second, **each outcome (ie, endpoint or intermediate) linked to a clearly stated hypothesis and intended to be analysed and reported should be adequately powered**. Adequate power allows the researcher to detect an impact of a magnitude that is both meaningful and feasible. Meaningful implies that an effect of this size is known to be associated with functional benefit or harm. Feasible is defined as being in line with our current understanding of what the (biological, social, psychological) effect size of the intervention on this outcome could be. For instance, an effect on birth weight of 500 g is meaningful but not feasible with typical nutrition and health interventions during pregnancy in women who are not severely undernourished; an effect of 70 g is both meaningful and feasible. Adequately powering studies has implications for sample size and thus cost. Consequently, researchers may not be able to adequately power the study for all relevant outcomes due to budget constraints. Including outcomes for which the trial is not adequately powered is common in trials of complex interventions but is analogous to a virologist hoping to detect a virus using a light microscope. Including underpowered outcomes is not recommended because inference for these outcomes will only be exploratory. Exceptions are when researchers expect the underpowered outcome (such as mortality) to be used later in a meta-analysis or where preliminary information is sought about the variability of an outcome. In these cases, descriptive information should be reported but no inferences should be made.

Third, before the trial is conducted, **researchers need to be explicit and fully transparent about the hypotheses and the outcomes**, however many, that they plan to evaluate and the tests they plan to conduct.^23^ A trial registration and statistical analysis plan are used for this purpose. Any underpowered outcomes should be transparently identified as such. Once the trial has been concluded, outcomes should be strictly and comprehensively reported as planned, regardless of whether an intervention effect was found. Additional unplanned outcomes and analyses need to be clearly identified, which requires transparency from investigators about which tests were done in addition to those that relate to a priori hypotheses. Investigators should thus disclose which comparisons were preplanned and which were not.

## Multiplicity in trials of complex interventions

Multiplicity occurs when many comparisons are carried out. Multiplicity has raised concerns about false-positive findings, which has led to recommendations to limit the number of comparisons made and/or to implement statistical adjustments based on the number of comparisons that are made. The latter recommendation is controversial with markedly opposing views.^40 41^ Multiplicity can arise in trials in general for six reasons:

1. A trial has multiple outcomes of interest.
2. Researchers conduct analyses to understand differences in effects across subgroups.
3. Trials have more than two study arms.
4. Findings from initial comparisons prompt examining other comparisons or relationships.
5. Analyses of repeated measures of the same outcomes are conducted, that is, when participants are assessed at multiple visits.
6. Interim analyses are conducted.

We discuss the first reason (ie, multiple outcomes) in depth and then each of the other five reasons briefly for trials of complex interventions.

### Multiple outcomes of interest

When a set of hypotheses (each about a different outcome) is tested simultaneously, the overall type I error rate, that is, the probability of wrongly rejecting at least one null hypothesis, increases.^40^ Simultaneous hypothesis testing assumes a universal null hypothesis that the intervention has no effect for all the outcomes investigated versus the alternative hypothesis of impact on at least one of these outcomes. Adjusting for multiplicity is only warranted if a set of hypotheses is tested simultaneously in this formal sense,^24 40^ but this test of a universal null vs the stated alternative hypothesis is nearly always irrelevant to the scientific or evaluation questions being investigated.^40 42^ When each hypothesis is limited to a single outcome, as is typically the case in impact evaluation, the probability of a false positive result remains the same irrespective of whether one or a million comparisons are tested.^40^ Because the p value is a simple transformation of a test statistic (eg, t-statistic or χ^2^ statistic) for a given outcome to a 0–1 scale using the assumed (eg, t or χ^2^) distribution, the type I error rate holds for that outcome regardless of whether other outcomes have been similarly transformed.

We illustrate this with the example of the Nutrition Embedded Evaluation Programme Impact Evaluation (NEEP-IE) trial in Malawi, which evaluated the impact of a complex intervention.^43^ The intervention was designed to improve child nutrition through several paths. First, the intervention was expected to increase agriculture production which would subsequently improve household-level availability of nutritious foods. Second, the nutrition behavioural change communication sought to improve diets and feeding practices by increasing caregiver knowledge. Third, by increasing the regularity and quality of meals in the community-based childcare centres (CBCC), the intervention was expected to promote CBCC participation and enhance learning and nutritional status.

The NEEP-IE trial had several main outcomes and corresponding hypotheses (online supplemental table 1). Multiplicity correction would have been required if the authors had tested the universal null hypothesis that NEEP-IE did not have an impact on any of these outcomes versus the alternative hypothesis that it had an impact on at least one outcome, that is, ‘the intervention increased agricultural production OR increased the diversity of agricultural production OR increased preschooler CBCC enrollment OR increased preschooler CBCC attendance OR increased child dietary intake’. This simultaneous hypothesis testing only makes sense if the researchers defined programme effectiveness as at least one of these five outcomes having changed meaningfully. This is, however, not how researchers generally draw conclusions about the effectiveness of complex interventions. The expectation that distinct, independent and perhaps alternative paths of impact are operating negates the utility of the universal null hypothesis. Even within a specific impact path, however, simultaneous hypothesis testing is not helpful to assess the effectiveness of the intervention. Instead, the impact of the intervention is first assessed for each of the individual study outcomes. The impact across all outcomes is then used to understand how and why the intervention improved some outcomes but not others. In the NEEP-IE study, the authors concluded that the intervention increased household production and diversity of production and had positive effects on sibling dietary diversity but did not increase CBCC enrollment or attendance. The authors did not make an overall (ie, intervention-wide) effectiveness statement. Instead, they used the study findings to identify why the programme was effective at improving some outcomes and not others.

10.1136/bmjgh-2022-008597.supp1 Supplementary data

Unnecessarily adjusting for multiplicity is costly. One direct consequence of decreasing type I errors (which is the objective of multiplicity adjustments) is that type II errors (ie, not identifying an impact when it exists) increase.^24 40 42^ The only solution is to increase the sample size, which has a financial and a societal (eg, economic, effort, time) cost, since more study participants are needed. Using more resources and enrolling more study participants than needed is unethical.^44^ Another direct consequence is that inferences may be inaccurate because in many situations outcomes are not independent as some multiplicity adjustment procedures assume,^40 42^ resulting in conservative, that is, biased tests.

Multiplicity concerns have led some to recommend combining several outcomes into a composite outcome measure,^13 14 26^ but composite outcome measures create interpretational challenges and often lack real-world relevancy.^40 45^ For example, the composite indicator of minimally acceptable diet for infants and young children is less useful for decision-makers, because of the difficulty to meaningfully interpret and act on it, than are the component indicators of minimal dietary diversity and minimal meal frequency.^46 47^ A positive effect on a composite outcome measure provides no information on which of the individual outcomes were affected. Likewise, a null effect on the composite outcome measure may hide a meaningful effect on one or several of the individual outcomes. Since complex intervention trials typically seek to understand how the intervention affected multiple outcomes, the use of composite outcomes measures is not recommended.

In summary, post hoc analytical adjustment for multiplicity resulting from multiple outcomes should not be done unless simultaneous hypothesis testing was planned, which is unlikely in a complex intervention trial.^24 40 42^ Careful selection of outcomes based on programme theory and having one measure per outcome (and not making a composite of measures) helps prevent an excess number of hypothesis tests.

### Subgroup analyses

Subgroup analyses often are undertaken to understand differences in effects across groups defined by baseline participant characteristics such as child age or household wealth.^41^ If no hypotheses about subgroups were planned, then the analyses are exploratory and should be identified as such with no need to adjust for multiplicity. If the subgroup analyses were planned, that is, hypotheses have been stated, then adjusting for multiplicity is not necessary if all planned subgroup analyses are reported and the interpretation of them is done carefully. Rather than adjusting for multiplicity, the investigator and readers should examine all p values or CIs of the set of subgroups analysed and assess the likelihood that the findings would be due to chance considering the number of subgroups analysed. An important concern is that subgroup analyses will often be inadequately powered and thus more likely to produce false-negative results, providing an additional justification why they should be considered exploratory. Furthermore, randomisation typically is not conducted by subgroup which means that the arms may not be equivalent for all participant characteristics at the beginning of the trial especially when subgroups are small. Within subgroups, differences in outcomes across arms may thus be due to pre-existing differences between participants. In sum, only when subgroup analyses are adequately powered and prespecified should the findings be considered confirmatory causal evidence.

### More than two study arms

Trials often have more than two study arms. Early in the last century, statisticians understood that doing a separate trial for each intervention was wasteful, inefficient and prevented investigating interactions.^48^ For example, the MINIMat RCT in Bangladesh had 12 arms formed by the combination of early vs usual food supplementation, three types of micronutrient supplements, and intensive versus usual lactation counselling in a factorial design.^49^ This design allowed the examination of all three types of interventions at the same time and the testing of the principal hypothesis which was that the synergistic combination of early food supplementation and multiple micronutrients would be superior to the other five combinations of nutrition interventions during pregnancy. Standard methods for optimally handling multiple comparisons arising from multiple study arms have been developed. The principles for these methods are to (1) plan the comparisons in advance, (2) keep the number of comparisons for a given outcome to one less than the total number of arms^48^ and (3) if these first two conditions do not hold then use a procedure optimised for the type of comparison (eg, Tukey test for all pairwise comparisons).^48^

### Initial comparisons prompt other comparisons

In any scientific study, findings from initial comparisons may prompt further examination of other comparisons or relationships. Whereas the initial comparisons ideally are preplanned, the comparisons that follow are not preplanned and must therefore be considered exploratory. For transparency, investigators should disclose which comparisons were preplanned and which were not, and how the decision to pursue further comparisons depended on the initial findings.

### Analyses repeated at multiple visits

When participants are assessed at multiple visits, analyses could be repeated with data from each of these visits. In trials of complex interventions, data from multiple visits are typically used to understand the unfolding of effects over time; analyses can also be done with all visits together. Investigators should decide and declare in advance in the trial protocol or analysis plan how the analyses will be conducted for each outcome. In some cases, the multiple measurements may be reduced to a single effect estimate such as the difference between the first and last visits or the linear trend over all visits. When multiple visits are to be used to estimate an overall effect, statistical procedures that appropriately account for the lack of independence among the visits must be employed.

### Interim analyses

Interim analyses are usually done to consider early stopping of trials to address ethical concerns about potential harms or when evidence of effectiveness is sufficient to not deprive participants in a comparison arm of demonstrated benefits. Accepted methods for conducting interim analyses have been established.^41^ Interim analyses are not usually done in trials of complex interventions.

## Other important features of trials for complex interventions

### Assessment of implementation of the intervention

For an intervention to be effective, the intervention components must be implemented with fidelity, reach participants, be delivered to participants, and be received by participants.^50–52^ Details about the experience, training and monitoring of implementers and the timing, intensity, fidelity, reach and dose of the interventions should be assessed through process evaluation and implementation research and reported. Measuring these implementation-related characteristics of interventions with multiple components and programme impact paths can be challenging. For example, an intervention may involve agricultural training, behaviour change communication, visits to health clinics, management of microcredit loans, and other activities. Specifying what is the adequate fidelity, reach, and dose across intervention components a priori in an intervention protocol is important to be able to understand the effectiveness of the intervention and to conduct analyses that attempt to account for less than full adherence to the intervention (see below).

### Selection bias and attrition

Selection bias arises when inherent differences between the individuals (or groups) assigned to trial arms result in biased effect estimates. Selection bias can arise at any stage of a field-based study from the initial recruitment through to completion of the data collection. Random assignment minimises (but does not eliminate) the potential for selection bias in the early stages of a study by making the trial arms comparable. Attrition bias may result from loss of participants during a study, which may be particularly problematic in trials of complex interventions, if attrition is large and/or differential between arms.

In trials of complex interventions, selection and attrition biases can be large and can result from multiple forces because people in the intervention arm are subject to the intervention protocol and people in the comparison arm are not. Assignment in complex intervention trials should be concealed before consent to participate is given whenever possible. Given the nature of complex interventions, blinding is typically not possible because individuals living in a village, for example, can become aware of the presence of the intervention. Agreement to participate in the trial should be obtained before random assignment at the cluster level; the intervention, however, may have started before individuals consent to being in the study. A cluster-randomised trial evaluating the impact on pregnancy outcomes, for instance, may enrol a new cohort of pregnant women each month. Participation in the data collection and adherence to the intervention, although theoretically distinct, can be intertwined. For example, participants in the intervention arm who agree to participate in the study or who adhere more to the intervention protocol can both be more motivated, or more in need, than those who do not agree or adhere less. Those more motivated or in need may be more likely to have data collected and/or to stay in the data collection. On the other hand, people who are in the intervention arm may find the intervention protocol burdensome and be more likely to drop out of the study.

Minimising selection and attrition and documenting the selection and retention processes are important. If possible, minimal data on participants who decline participation during the recruitment and enrolment stages should be collected for comparison with study participants. After assignment and throughout the follow-up and data collection periods, procedures should be the same in all arms to the extent possible. Although participants assigned to a complex intervention may engage with only some intervention components or only some of the time, they should be followed in the data collection regardless of receipt of or engagement with the intervention.^53^ To help assess the consequences of attrition, minimal data collection with those who drop out may be possible, even if full data collection cannot occur for those otherwise lost to attrition.^54^

### Decision on the type of analysis

Although the validity of the counterfactual is determined by the study design and its execution, the type of analysis that is used affects how well the trial provides a valid counterfactual for the intervention arm. The primary types of analysis are intent-to-treat and per-protocol, with protocol referring to the intervention and not to the data collection or analysis

The intention-to-treat analysis estimates the average impact of the offer of the intervention on outcomes, rather than the impact of participation in the intervention. Since many study participants may not fully adhere, or adhere at all, to the intervention protocol, the estimates of effectiveness may be lower than the efficacy of the intervention, that is, what effects would have been achieved if the fidelity, reach, dose delivered, and dose received were ideal. Intent-to-treat means that all eligible participants regardless of adherence with the intervention protocol should be included in the analysis of results whenever possible, in contrast to per-protocol which is an analysis of only those who adhered to or fully took up the intervention protocol.^55^ Collecting data on all who were included initially in all arms allows for comparison of those who were assigned to the intervention activities (regardless of fidelity, reach, dose delivered to them or dose received by them) with those who did not. Comparison of completers and drop-outs from the data collection in all arms helps to assess attrition bias. In practice, fully achieving an intent-to-treat analysis is often difficult because of missing data resulting from attrition if the study follows individuals. The intent-to-treat analysis is thus conducted on participants for which the outcome is known. Attrition is documented in each arm.

A per-protocol (ie, as-treated or treatment-on-treated) analysis restricts the analytical sample in the intervention arm to those who fully adhered to the intervention protocol, which needs to be specified prior to the analysis.^56^ Measuring adherence in trials of complex interventions, however, is difficult because of differences in adherence to the multiple components of the intervention.

Since study participants who fully adhered to the intervention protocol may have been more motivated or able to adhere, the estimates of effectiveness may be biased because those fully adherent are self-selected. Sometimes per-protocol analyses are modified to include those with partial adherence to try to balance the possible underestimation of the potential intervention effects that comes from an intent-to-treat analysis when there is less than full adherence and the bias that comes from restricting the analytic sample to only those fully adherent to the intervention protocol.^57^ An alternative method to account for less than full adherence is the use of instrumental variables to estimate the local average treatment effect, that is, the effect of the intervention for those who adhered to it.^1 58 59^ Another type of analysis is the dose–response analysis, where participants are classified based on the level of adherence or exposure to the intervention. This analysis is used to understand if observed effects are associated with participant adherence. It has the same associated biases described above.

The CONSORT statement indicates that intention-to-treat is the preferred analysis type and acknowledges that performing strict intent-to-treat analysis is challenging.^7^ An intention-to-treat analysis should always be reported for at least three reasons. First, it takes full advantage of the random assignment to establish a valid counterfactual. Second, if the intention-to-treat results show that the intervention had effects despite having people in the analytic sample who did not fully adhere to the intervention protocol, then the results provide strong evidence of effectiveness even if it is an underestimate of the intervention’s (biological or behavioural) efficacy. Third, the intention-to-treat estimates reflect what policy makers, funders, and programme implementers can reasonably expect the average population impact to be when the programme is implemented at scale. The estimates of intention-to-treat effects thus may be more relevant for policy than estimates of efficacy. A per-protocol or instrumental variable analysis may provide useful information in addition to the intention-to-treat analysis but should be considered as a secondary analysis designed to help clarify why, or why not, an intervention had its observed effect.

## Recommendations for RCTs of complex interventions

The purpose of trials of complex interventions is to evaluate how different outcomes were affected, how the multiple components that make up the intervention worked together, and what the population-level impact of the intervention may be. To maximise what can be learnt from each trial, we recommend the following steps when designing and implementing RCTs of complex interventions. Many of our recommendations are applicable to all RCTs.

### Step 1: specify the programme theory

The programme theory (also referred to as theoretical model, theory of change, causal paths or paths to impact), which identifies the key intervention components and how they are expected to be linked to achieve impact, is designed by the group of programme implementers, evaluators and other relevant stakeholders. Displaying the programme theory in a programme impact path diagram is an essential exercise for recording the programme theory.^60 61^ The programme impact path diagram should specify both the intervention actions and the changes in the targeted participants that are together expected to generate benefits.^62^

### Step 2: identify outcomes and measures

Using the programme impact paths, all outcomes that the intervention is designed or expected to affect are identified. The list of outcomes should include those that represent the end and intermediate points along the impact paths, both desirable and undesirable, and should include both what implementers and participants do (ie, actions they are expected to take) and what changes are meant to occur. Primary outcomes (ie, relevant based on the intervention intent and programme theory) should be identified. These outcomes will be accompanied by hypotheses and be adequately powered (step 3). Trials of complex interventions are likely to have many primary outcomes.

Once all outcomes have been identified, measures that reflect these outcomes need to be chosen. The choice of outcome measures is determined by validity, measurement feasibility and cost. Ideally one measure per outcome is chosen. Sometimes multiple questionnaire items are intended to be combined to form a scale to measure an outcome.

### Step 3: calculate sample size requirements for adequate power

Sample size calculations determine the number of observations needed to detect the minimum meaningful effect of the intervention on each of the identified primary outcome measures. For trials that cannot be easily repeated such as those typically conducted to evaluate complex interventions 80% power which is frequently used in trials may be too low, and we recommend that at least 90% power is used. The outcome with the highest sample size requirement will determine the overall study sample size. Finally, additional sample size provisions need to be made for planned subgroup analyses and planned tests of effect modification as well as attrition, missing data and any other problem that will reduce the number of observations that can be included in the data analysis.^38^ Sample size requirements for some outcomes may be beyond the study’s budget. These secondary outcomes must be identified as inadequately powered; although they can be assessed, inference will be only exploratory (table 2).

### Step 4: register the trial and produce the statistical analysis plan

In the trial registration (online supplemental table 2), which should be completed before the trial is started, investigators declare the outcomes that they will analyse, and which outcomes are adequately powered. The statistical analysis plan should provide details about (1) administration of the study; (2) background, rationale and objectives; (3) study methods; (4) statistical principles; (5) trial population and (6) planned analytical methods.^63^ The statistical analysis plan is an agreement among the principal investigators, possibly other investigators, stakeholders or sponsors, the senior statistician and data analysts. It should be completed and registered before analysis begins. It should be detailed enough for other analysts to use the data, conduct the analysis, and arrive at the same results. Any deviations from the analysis plan should be documented. The study protocol, statistical analysis plan, and trial reports and publications need to distinguish between primary outcomes, that is, those that are declared (ie, registered) and adequately powered, and all other outcomes (table 2).

### Step 5: assess programme implementation

All activities to standardise the interventions across participants should be described. Variation in the timing, intensity, fidelity, reach and dose of the delivered interventions should be fully documented through a thoroughly designed process evaluation.

### Step 6: focus on declared and adequately powered outcomes and limit the inference for undeclared and inadequately powered outcomes

Confirmatory causal inference is limited to the primary outcomes, that is, the outcomes that were declared and registered before the trial was started and for which the study was adequately powered (table 2). Inference about outcomes that were not declared before the trial started should be considered exploratory because having clarity on how and when decisions were made to test these outcomes is difficult. The interpretation of the absence of an effect depends on statistical power. When no meaningful and feasible effect is observed and the trial was adequately powered, we can conclude (with a quantifiable level of confidence) that the trial did not have an effect of the magnitude for which the trial was powered for that outcome. When a trial was not powered to detect a feasible and meaningful effect, however, a finding of no effect could be because either the effect is truly absent, or the effect was too small to be detected. In this case, researcher should avoid interpreting these results as the programme not having an effect.

### Step 7: maintain transparency when reporting outcomes

The disposition of participants should be clearly documented through a CONSORT flow diagram.^7^ If the trial used cluster randomization, the diagram should report on the disposition of the clusters and individual participants by arm, including consent and enrolment rates.

All planned outcomes should be reported irrespective of whether an intervention effect was found, and all unplanned outcomes and analyses need to be identified. For each outcome, an estimate of effect (even if small or null) and the confidence in that estimate (ie, exact p value or CI) are reported without adjustment for multiplicity of outcomes so that readers can interpret what was found for each outcome.

Even when outcomes are declared and registered before the trial, researchers often only report on those outcomes that the intervention affected. Although reporting all primary outcomes in one paper could eliminate this problem of reporting bias, trials may have more primary outcomes than can be accommodated in a single journal manuscript. Furthermore, collaborating researchers may want to report the impact on specific outcomes in different disciplinary journals. We recommend the mandatory inclusion in each trial paper of an overview of all primary and secondary outcomes as declared in the trial registration, the outcomes analysed and reported in the paper, references to papers for previously reported outcomes, and outcomes that remain to be analysed and reported. The proposed recommendation will increase transparency and provide a direct quantifiable measure of potential publication bias, that is, the extent to which researchers report on some (but not all) of the registered outcomes. Journal editors and reviewers are important for ensuring transparency by thoroughly reviewing submitted trial manuscripts. Their review should include carefully comparing the reported outcomes against the outcomes that were registered, assessing whether outcomes were powered and checking whether the authors followed the statistical analysis plan.

## Conclusion

Complex interventions with multiple components from multiple sectors can play an important role in addressing problems such as poverty, undernutrition, poor child development and health. These interventions typically address several determinants of the problem through multiple paths. Rigorous evaluations of complex interventions are needed to strengthen the evidence base on how to improve effectiveness. In this paper, we provided guidance on how to ensure that RCTs to assess the impact of these complex interventions meet high scientific standards while maximising the evidence that can be obtained.

## Data Availability

No data are available.
