# Supplementary material for: Strengthening causal inference from randomised controlled trials of complex interventions
Source: BMJ Glob Health. 2022 Jun 9;7(6):e008597. doi: 10.1136/bmjgh-2022-008597 (PMC9189821; doi:10.1136/bmjgh-2022-008597)
Supplement: Supplementary data [file bmjgh-2022-008597supp001.pdf]

Supplemental material

Supplemental Table 1: The NEEP-IE trial's main outcomes and hypotheses (simplified)

**Outcome**	**Null hypothesis (H_0)**	**Alternative hypothesis (H_1)**
Household agricultural production	The NEEP-IE intervention did not increase household agricultural production (quantity)	The NEEP-IE intervention increased household agricultural production (quantity)
Household agricultural production diversity	The NEEP-IE intervention did not increase household agricultural production (diversity)	The NEEP-IE intervention increased household agricultural production (diversity)
Preschooler enrollment in the community-based childcare centers	The NEEP-IE intervention did not increase enrollment in the community-based childcare centers	The NEEP-IE intervention increased enrollment in the community-based childcare centers
Preschooler attendance at the community-based childcare centers	The NEEP-IE intervention did not increase attendance in the community-based childcare centers	The NEEP-IE intervention increased attendance in the community-based childcare centers
Child dietary intake	The NEEP-IE intervention did not improve child dietary intake	The NEEP-IE intervention improved dietary intake
